# Insertion torque, flexural strength and surface alterations of stainless steel and titanium alloy orthodontic mini-implants: an in vitro study

**DOI:** 10.1590/2177-6709.29.2.e2423282.oar

**Published:** 2024-05-20

**Authors:** Gustavo Lopes PULS, Guido Artemio MARAÑÓN-VÁSQUEZ, Christian Andrew Vargas RAMOS, Caio Luiz Bitencourt REIS, Andréa Cândido dos REIS, Maria Bernadete Sasso STUANI, Fábio Lourenço ROMANO, Mírian Aiko Nakane MATSUMOTO

**Affiliations:** 1 Universidade de São Paulo, Faculdade de Odontologia de Ribeirão Preto, Departamento de Odontopediatria (Ribeirão Preto/SP, Brazil).; 2 Universidade de São Paulo, Faculdade de Odontologia de Ribeirão Preto, Especialização em Ortodontia (Ribeirão Preto/SP, Brazil).; 3 Universidade de São Paulo, Faculdade de Odontologia de Ribeirão Preto, Departamento de Materiais Odontológicos e Prótese (Ribeirão Preto/SP, Brazil).

**Keywords:** Torque, Flexural strength, Stainless steel, Titanium alloy (Ti-6Al-4V), Orthodontic appliances, Torque, Resistência à flexão, Aço inoxidável, Liga de titânio (Ti-6Al-4V), Aparelhos ortodônticos

## Abstract

**Objective:**

This study aimed to compare the insertion torque (IT), flexural strength (FS) and surface alterations between stainless steel (SS-MIs) and titanium alloy (Ti-MIs) orthodontic mini-implants.

**Methods:**

Twenty-four MIs (2 x 10 mm; SS-MIs, n = 12; Ti-MIs, n = 12) were inserted on artificial bone blocks of 20 lb/ft^3^ (20 PCF) and 40 lb/ft^3^ (40 PCF) density. The maximum IT was recorded using a digital torque meter. FS was evaluated at 2, 3 and 4 mm-deflection. Surface topography and chemical composition of MIs were assessed by scanning electron microscopy (SEM) and energy dispersive X-ray spectroscopy (EDS). General linear and mixed models were used to assess the effect of the MI type, bone density and deflection on the evaluated outcomes.

**Results:**

The IT of Ti-MIs was 1.1 Ncm greater than that obtained for the SS-MIs (*p*= 0.018). The IT for MIs inserted in 40 PCF test blocks was 5.4 Ncm greater than that for those inserted in 20 PCF test blocks (*p* < 0.001). SS-MIs inserted in higher density bone (40 PCF) had significantly higher flexural strength than the other groups, at 2 mm (98.7 ± 5.1 Ncm), 3 mm (112.0 ± 3.9 Ncm) and 4 mm (120.0 ± 3.4 Ncm) of deflection (*p*< 0.001). SEM evidenced fractures in the Ti-MIs. EDS revealed incorporation of 18% of C and 2.06% of O in the loaded SS-MIs, and 3.91% of C in the loaded Ti-MIs.

**Conclusions:**

Based on the findings of this *in vitro* study, it seems that SS-MIs offer sufficient stability and exhibit greater mechanical strength, compared to Ti-MIs when inserted into higher density bone.

## RESUMO

**Objetivo:**

O objetivo deste estudo foi comparar o torque de inserção (TI), a resistência flexural (RF) e as alterações de superfície em mini-implantes ortodônticos de aço inoxidável (MIs-Ai) e de liga de titânio (MIs-Ti).

**Métodos:**

Vinte e quatro MIs (2 x 10 mm; MIs-Ai, n = 12; MIs-Ti, n = 12) foram inseridos em blocos de osso artificial de densidades de 20 lb/ft^3^ (20 PCF) e 40 lb/ft^3^ (40 PCF). O torque máximo de inserção foi registrado por meio de um torquímetro digital. A resistência flexural foi avaliada nas deflexões de 2, 3 e 4 mm. Topografia de superfície e composição química dos MIs foram avaliadas por Microscopia Eletrônica de Varredura (MEV) e Espectroscopia de Energia Dispersiva de Raios X (EDS). Modelos lineares gerais e mistos foram utilizados para avaliar o efeito do tipo de MI, da densidade óssea e da deflexão nos desfechos avaliados.

**Resultados:**

O TI dos MIs-Ti foi 1,1 Ncm maior do que o obtido para os MIs-Ai (P = 0,018). O TI para MIs inseridos em blocos de teste de 40 PCF foi 5,4 Ncm maior do que para aqueles inseridos em blocos de teste 20 PCF (*p* < 0,001). MIs-Ai inseridos em osso de maior densidade (40 PCF) apresentaram resistência flexural significativamente maior do que outros grupos, em deflexões de 2 mm (98,7 ± 5,1 Ncm), 3 mm (112,0 ± 3,9 Ncm) e 4 mm (120,0 ± 3,4 Ncm) (*p* < 0,001). A MEV evidenciou fraturas nos MIs-Ti. A EDS revelou incorporação de 18% de C e 2,06% de O nos MIs-Ai e 3,91% de C nos MIs-Ti, ambos submetidos a testes mecânicos.

**Conclusões:**

Com base nos resultados desse estudo *in vitro*, os MIs-Ai aparentam oferecer adequada estabilidade e maior resistência mecânica, em comparação aos MIs-Ti, quando inseridos em osso de maior densidade.

## INTRODUCTION

Orthodontic mini-implants (MIs) are devices that provide temporary anchorage for the application of various orthodontic mechanics without the need for patient collaboration.^1^ ^-^ ^3^ To achieve optimal clinical performance when using MIs, these devices should be made of a material whose mechanical properties allow them to provide adequate stability to support immediate loads without suffering long-term alterations.

MIs are commonly made of titanium alloy (Ti-MIs; Ti-6Al-4V) or austenitic stainless steel (SS-MIs; AISI 316L).^4^ Current evidence seems to show that the MI’s material would not be a determining factor in achieving clinical success using these devices.^5^ Similar success rates and histological responses have been reported for both types of MIs.^5^ ^-^ ^8^ Therefore, both are suitable for orthodontic use. However, in certain clinical contexts where there is a greater bone density and thickness at the insertion site (i.e., extra-alveolar regions), it would be interesting to choose MIs that provide greater mechanical resistance and, consequently, a lower risk of fracture. Thus, SS-MIs are usually recommended for extra-alveolar use instead of Ti-MIs, due to their greater toughness.^9^ ^,^ ^10^

Unfortunately, the literature on the differences in mechanical properties between Ti-MIs and SS-MIs is controversial. A previous study showed a higher insertion torque for SS-MIs,^6^ while others demonstrated, through torque analyses and/or resonance frequency analysis, similar stability values for both types of MIs.^11^ ^,^ ^12^ Although greater flexural and torsional strength has been reported for SS-MIs,^13^ there is also research demonstrating equal mechanical resistance between Ti-MIs and SS-MIs.^14^ Regarding surface deformation, inconsistent results were also observed. While a study reported a higher frequency of deformation in Ti-MIs,^15^ another investigation did not show important morphological damage in the threads of any type of MI.^13^

Since the evidence on the matter is still limited and inconclusive, new research is necessary to confirm or reject previous findings. Therefore, the present study aimed to provide further information on the topic, comparing the insertion torque, flexural strength and surface alterations between SS-MIs and Ti-MIs inserted in artificial bone of different densities.

## MATERIAL AND METHODS

This *in vitro* study was conducted and reported following the Checklist for Reporting *in vitro* Studies (CRIS) guidelines.^16^

### INDEPENDENT VARIABLES ASSESSED

The independent variables evaluated in the present study were the type of MI (SS-MIs and Ti-MIs) and the density of the artificial bone. For the flexural strength evaluations, the variable degree of deflection was also evaluated.

**Figure 1:**
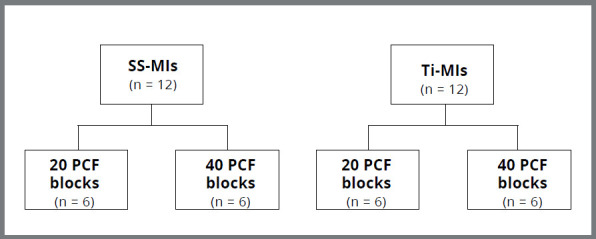
Distribution of the study groups according to the mini-implant type and density of the artificial bone. SS-MIs = stainless steel mini-implants, Ti-MIs = titanium alloy mini-implants.

A total of 24 MIs of 2 x 10 x 4 mm (diameter x length x transmucosal profile), made of stainless steel (n = 12; Morelli, Sorocaba/SP, Brazil) or titanium alloy (n = 12; Peclab, Belo Horizonte/MG, Brazil) were inserted on mechanical test blocks of artificial bone measuring 2 x 2 x 3 cm (length x width x height). Synthetic bone models constructed from solid rigid polyurethane foam (Nacional Ossos, Jaú, SP, Brazil) of 20 lb/ft^3^ (20 PCF; 0.32 g/cm^3^) and 40 lb/ft^3^ (40 PCF; 0.64 g/cm^3^) density were chosen as bone tissue equivalent for the present study. Solid rigid polyurethane foam has been recognized as a standard for testing orthopedic devices and instruments, including bone screws, due to its adequate representation of adult human bone (ASTM F-1839-08). The selection of the densities used, as bone equivalents of lower (0.32 g/cm^3^) and higher (0.64 g/cm^3^) quality, was based on a previous study.^17^ According to the type of MI and density of the artificial bone, four study groups (n = 6 each) were finally defined, as shown in Figure 1.

### OUTCOMES (DEPENDENT VARIABLES) ASSESSED

The dependent variables evaluated in the present study were insertion torque, flexural strength, surface topography and chemical composition.

**Figure 2:**
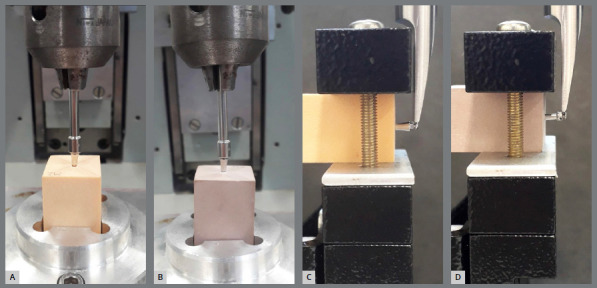
Mechanical tests. **A,** B) insertion of mini-implants in test blocks of 20 PCF and 40 PCF, respectively, for measurement of insertion torque. **C,** D) Application of perpendicular load on the head of the mini-implants for measurement of flexural strength.

Pilot holes of 1-mm depth were performed in the center of the test blocks, prior to the insertion of MIs, using a 1.0-mm diameter twist drill (Conexão Sistemas de Prótese, Arujá/SP, Brazil). As previously described,^18^ the MIs were inserted with a specific manual key for each type of MI, connected to a digital torque meter (model TQ-8800; Lutron, Taipei, Taiwan). Using a mechanical support, the insertion was carried out perpendicularly until all the threads of MIs were completely inside the artificial bone (Fig. 2A and 2B). The maximum insertion torque was recorded with a precision of 0.1 Newton-centimeter (Ncm). After the installation, the MIs received load on their head, perpendicular to their longitudinal axis, at a speed of 0.5 mm/min, and load of 50 Kgf using a Universal Testing Machine mBio (Biopdi, São Carlos/SP, Brazil) (Fig. 2C and 2D). Flexural strength at 2, 3 and 4-mm deflection was recorded (Ncm). 

One MI per group, randomly selected, and a new MI (not submitted to mechanical loading) for each MI type, were used for surface topography and chemical composition analyses by means of scanning electron microscopy (SEM) and energy dispersive X-ray spectroscopy (EDS), respectively. The MIs were fixed on metallic platforms with the aid of colloidal graphite. An X-ray detector system coupled to a scanning electron microscope (JSM-6610LV, JEOL, Akishima, Japan), operating at 20kv, was utilized. The SEM Control User Interface program v. 3.06 was used to acquire photomicrographs of the surface of the head and the middle third of the MIs, with magnification of 30x. Higher magnifications were used to show characteristics of the observed failures. Surface characteristics were evaluated in a qualitative manner. The chemical composition was analyzed at the same sites, as the surface topography evaluation. The Oxford Aztec software (version 3.3) was used to obtain the relative amounts (%) of the chemical components of the region of interest.

### SAMPLE SIZE

To estimate the sample size, an *a priori* calculation was performed for pairwise comparisons of independent samples (two-tailed *t*-test) based on previous reported results on the insertion torque (Ncm) of SS-MIs (4.4 ± 0.56) and Ti-MIs (7.5 ± 0.79).^12^ These values resulted in an effect size d = 4.53, which was used for the subsequent calculations. The estimates were made in G*Power 3.1 software, considering the following parameters: effect size d = 4.53, α error probability = 0.05, power (1-β error probability) = 0.8, and allocation ratio = 1. The calculation resulted in a minimum sample size of three MIs per group. Considering the possibility of using non-parametric statistics, the estimated amount was doubled, resulting in six MIs by group.

Considering the limitations of the aforementioned approach, *post-hoc* calculations of the power achieved by the finally implemented statistical models were additionally performed. The power estimate for the general linear model was based on an effect size f = 2.89 (calculated based on η^2^p = 0.893, obtained from the implemented model), an α error probability = 0.05, total sample size = 24, numerator df = 1 and number of groups = 4. The power estimate for the mixed model was based on an effect size f = 10.49 (calculated based on η^2^p = 0.991, obtained from the implemented model), an α error probability = 0.05, total sample size = 24, number of groups = 4, number of measurements = 3, correlation among repeated measures = 0.5 and nonsphericity correction = 1.

### RANDOMIZATION AND BLINDING

The MIs were coded and randomized for each study group using a random sequence generator (https://www.random.org). This procedure was carried out by a researcher who did not participate in the MIs insertion procedures or in the measurements of the outcomes.

Blinding was not possible for any of the phases of the research.

### STATISTICAL METHODS

Descriptive statistics were used to present the data of the evaluated outcomes. A general linear model was implemented to evaluate the effect of the MI type (SS-MI/TI-MI), bone density (20 PCF/40 PCF), and the interaction MI type*Bone density on the insertion torque. Furthermore, to evaluate the effects on flexural strength, a mixed model was implemented, in which the MI type, bone density, deflection and the possible interactions (i.e., MI type*Bone density, MI type*Deflection, Bone density*Deflection, and MI type*Bone density*Deflection) were considered as fixed effects of variation, and the mini-implants were considered as a random intercept in the models. *Post-hoc* comparisons between the study groups using the Bonferroni test were carried out, in case of significant effects of the interactions were detected. Test assumptions were verified using the Shapiro-Wilk test to assess normality of residuals and Levene’s test to assess homogeneity of residual variances. All tests were performed in Jamovi software (version 2.0), using a significance level of 5%.

## RESULTS

### NUMBERS ANALYZED

No losses were reported during the evaluations; therefore, all 24 MIs were part of the analyses.

### OUTCOMES AND ESTIMATIONS

The EDS analysis confirmed the chemical composition of the MIs used in the present study. The new SS-MIs were made of Fe, Cr, Ni, Mo and Mn; while the new Ti-MIs contained Ti, Al and V (Table 1).

**Table 1:** Relative amounts (%) of chemical components in each mini-implant ( MI ) type.

Mini-implant (MI) type	Chemical component	New MIs	MIs inserted in 20 PCF blocks	MIs inserted in 40 PCF blocks
Stainless steel (SS-MIs)	C	---	18.72	---
	O	---	2.06	---
	Cr	18.12	14.71	18.49
	Mn	1.93	1.45	1.95
	Fe	62.13	49.45	62.17
	Ni	14.56	11.41	14.31
	Mo	2.56	2.20	3.08
Titanium alloy (Ti-MIs)	C	---	---	3.91
	Al	5.91	5.62	5.64
	Ti	90.30	90.42	86.73
	V	3.79	3.95	3.71

No significant effect of the interaction MI type*Bone Density on the insertion torque was detected (*p*= 0.565; Table 2). The MI type (*p*= 0.018) and bone density (*p* < 0.001) had significant independent effects on the insertion torque values (Table 2). The insertion torque of the TI-MI was 1.1 Ncm greater than that obtained for the SS-MI. The insertion torque for MIs inserted in 40 PCF test blocks was 5.4 Ncm greater than that for those inserted in 20 PCF test blocks. Detailed insertion torque values are reported in Table 3. The power of the model was greater than 90% for the sample size used.

**Table 2:** Effect of the variables MI type, bone density and interaction on the insertion torque values.

Variables	F	*p* **-value**
MI type	6.6	0.018*
Bone density	159.1	<0.001*
MI type*Bone density	0.3	0.565

* Indicate statistically significant effect.

**Table 3:** Means ± SD and mean differences (95% CI) of insertion torque according to the mini-implant type and bone density.

Variable	Group	Sample size	Mean ± SD	Mean difference (95% CI)
MI type	Stainless steel (SS-MIs)	12	13.4 ± 3.0	1.1 (0.3 - 1.9)
	Titanium alloy (Ti-MIs)	12	14.5 ± 3.0	
Bone density	20PCF	12	11.3 ± 0.8	5.4 (4.6 - 6.2)
	40PCF	12	16.6 ± 1.4	

SD = standard deviation, CI = confidence interval.

**Figure 3:**
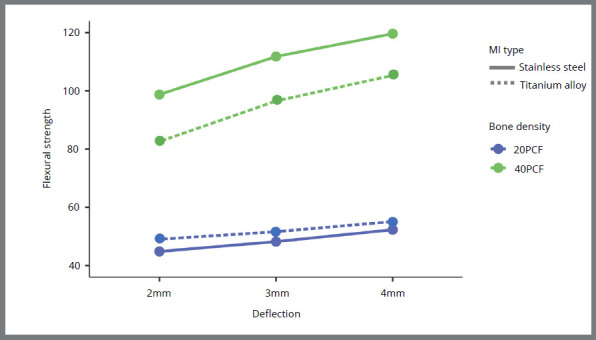
Flexural strength according to the MI type, bone density and deflection.

The interaction MI type*Bone Density*Deflection showed a significant effect on the flexural strength values (*p*= 0.021, Table 4). As expected, in general, the greater the degree of deflection, the greater the flexural strength. A significant effect of the interaction Bone density*Deflection was demonstrated (*p*< 0.001, Table 4) - that is, the increase in flexural strength as the deflection was greater was only evident in the MIs inserted in the 40 PCF test blocks, and not in the MIs inserted in the 20 PCF test blocks (Fig 3). A significant effect of the interaction Mini-implant*Bone density was also detected (*p*< 0.001, Table 4) - that is, the differences observed between SS-MIs and Ti-MIs were only evident in the MIs inserted in the 40 PCF blocks, and not in those inserted in the 20 PCF blocks (Fig 3). *Post-hoc* comparisons showed that SS-MIs inserted in higher density bone (40 PCF) had significantly higher flexural strength than the other groups, at 2 mm (98.7 ± 5.1 Ncm), 3 mm (112.0 ± 3.9 Ncm) and 4 mm (120.0 ± 3.4 Ncm) of deflection. The flexural strength values for all the groups evaluated are reported in Table 5. The power of the model was greater than 90% for the sample size used.

**Table 4:** Effect of the variables MI type, bone density, deflection and interactions on the flexural strength values.

Variables	F	***p*-value**
MI type	27.4	<0.001*
Bone density	2080.0	<0.001*
Deflection	1336.5	<0.001*
MI type*Bone density	66.8	<0.001*
MI type*Deflection	0.1	0.914
Bone density*Deflection	387.1	<0.001*
MI type*Bone density*Deflection	4.3	0.021*

* Indicate a statistically significant effect.

**Table 5:** Mean ± SD of flexural strength, according to MI type*bone density and deflection.

Group	Sample size (n)	Deflection		
		2 mm	3 mm	4 mm
		Mean ± SD	Mean ± SD	Mean ± SD
Stainless steel*20 PCF	6	44.8 ± 4.1^a^	48.2 ± 2.4^a^	52.3 ± 3.3^a^
Titanium alloy*20 PCF	6	48.9 ± 1.2^a^	51.5 ± 1.4^a^	55.0 ± 1.6^a^
Stainless steel*40 PCF	6	98.7 ± 5.1^b^	112.0 ± 3.9^b^	120.0 ± 3.4^b^
Titanium alloy*40 PCF	6	82.5 ± 2.2^c^	96.4 ± 1.6^c^	105.0 ± 1.6^c^

SD = standard deviation.

Different superscript letters indicate statistically significant difference among the values for the columns.

**Figure 4:**
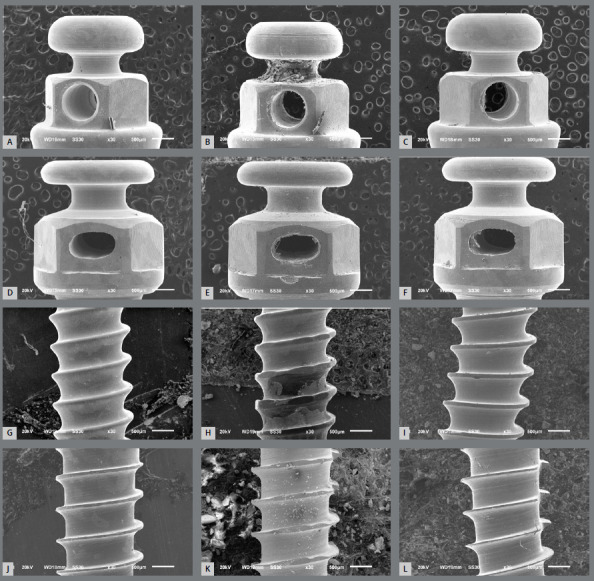
SEM analysis. **A, B, C, G, H, I**) stainless steel mini-implants; **D, E, F, J, K, L**) titanium alloy mini-implants; **A, D, G, J**) control (new) mini-implants (not submitted to mechanical loading); **B, E, H, K**) mini-implants installed in 20 PCF test blocks and submitted to loading; **C, F, I, L**) mini-implants installed in 40 PCF test blocks and submitted to loading.

**Figure 5:**
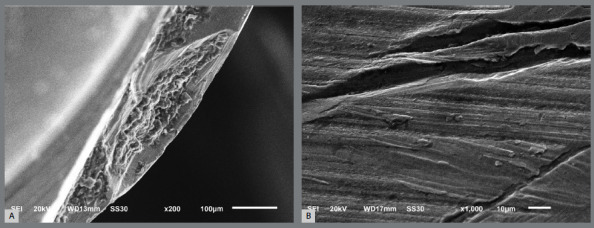
Surface alterations evidenced in titanium alloy mini-implants. A) microfracture, B) fracture lines.

SEM analysis showed no or minimal surface alteration in any of the MIs’ heads after being submitted to mechanical loading (Fig 4). The head surfaces of the MIs were homogeneous, well-polished and with minimal structural defects, such as striations. On the other hand, plastic deformation without fracture was observed in the threads of SS-MIs (Fig 4H and 4I), while obvious fractures on the middle third of the screw were observed in the threads of Ti-MIs (Fig 4K and 4L). Figure 5 shows, at higher magnification, a microfacture and oblique fracture lines in the Ti-MIs.

The EDS analysis evidenced the presence of 18% of C and 2.06% of O in the SS-MIs inserted in test blocks of 20 PCF, after being submitted to loading. Incorporation of 3.91% of C was also observed in the Ti-MIs placed in 40 PCF blocks. The percentages of the chemical components on the surface of the evaluated MIs are presented in Table 1.

## DISCUSSION

Although SS-MIs are recommended for extra-alveolar use due to their greater toughness,^9^ ^,^ ^10^ the literature is still controversial regarding the mechanical advantages of this type of MI, compared to Ti-MIs. To corroborate the available evidence, the present study aimed to compare primary stability assessed by means of the insertion torque, and flexural strength between SS-MIs and Ti-MIs, simulating a clinical context of force application in bone of different densities. The results demonstrated that: (a) primary stability is mainly influenced by the density of the bone and to a lesser extent by the MI’s material; (b) in higher density bones, the SS-MIs present greater flexural strength and less surface deformation than Ti-MIs.

Insertion torque is a parameter that reflects the frictional resistance between the screw and the surrounding bone, and is a widely used measure to evaluate mechanical stability.^19^ ^,^ ^20^ Although with low certainty of the evidence, the orthodontic literature is somewhat consistent about the existence of a positive correlation between the primary stability of the MIs and the quality of the receptor bone site (i.e., cortical/compact bone thickness).^2^ ^,^ ^21^ The findings of the present study confirmed this information. Regardless of the MI material, the insertion torque values were significantly higher when the MIs were inserted into more compact bone blocks (i.e., 40 PCF). 

Regarding the influence of the type of material, previous evidence comparing SS-MIs and Ti-MIs showed no or very little difference in stability values.^6^ ^,^ ^11^ An animal study assessing MIs of 6 x 1.6 mm reported that SS-MIs required significantly greater insertion torque than Ti-MIs; however, the values for each type of MI were not very distant from each other (SS-MIs: 12.00 ± 0.25 Ncm; Ti-MIs: 11.01 ± 0.24 Ncm).^6^ An *in vitro* study evaluating MIs of 10/12 x 2 mm inserted in artificial bone, but assessing primary stability by means of resonance frequency analysis, reported similar stability values for both MIs types.^11^ The findings of the present study also showed only a small difference in insertion torque values between both types of materials. There was even a trend for SS-MIs to show slightly lower values than Ti-MIs (SS-MIs: 13.40 ± 3.00 Ncm; Ti-MIs: 14.50 ± 3.00 Ncm). This would be in favor of SS-MIs, since it has been suggested that excessive stress could cause necrosis and local ischemia, which could prevent adequate secondary stability.^22^ ^,^ ^23^ Despite these findings, and evaluating the available evidence as a whole, it could be assumed that this difference seems not to be clinically relevant: both types of MI would show adequate primary stability when inserted in bone of greater or lesser density.

Flexural strength is the stress that exists at failure in bending. It is desired that the MIs have high flexural strength to avoid fracture of the MIs, mainly in contexts where the MIs are placed in higher density bone.^9^ ^,^ ^10^ A previous study evaluating 8-mm MIs demonstrated greater flexural strength for SS-MIs than for Ti-MIs.^13^ Another research carried out with extra-alveolar MIs of 10 and 11 mm showed no difference in the flexural resistance between both types of material.^14^ However, this study applied the bending load in a MI’s region close to the insertion surface and far from the head of the MIs, and evaluated 0.1 and 0.2 mm-deflections, favoring more similar results between SS-MIs and Ti-MIs. Both studies conducted their evaluations using standard mechanical testing procedures, in which MIs are fixed to rigid supports to receive bending loading. With the objective of simulating a more real clinical situation, where the MIs must resist bending in a biological tissue that is not completely rigid (i.e., MIs displace within the bone),^24^ the bending loads were applied with the MIs fixed in artificial bone of different densities. As expected, in lower density bone (i.e., 20 PCF), both types of MI showed similar flexural strength; the less dense bone initially yields to the loading application, the MI displaces, and a minor deformation of the MIs is observed. In the bone of greater density (i.e., 40 PCF), bone yields to a lesser extent, more load is necessary to displace the MIs and, consequently, greater deformation of the MIs is observed. In this last context, the SS-MIs showed better mechanical behavior. More force was necessary to achieve 2, 3, and 4 mm-deflections for the SS-MIs than for the Ti-MIs. The lower mechanical resistance of the Ti-MIs was evidenced by the presence of fractures close to the bending region of these MIs.

The above-mentioned results have an important clinical relevance, since they suggest that in higher density bone regions, SS-MIs would have greater mechanical resistance than Ti-MIs. Previous evidence has suggested that a bone density of 0.32 g/cm^3^ is mainly observed in the posterior region of the maxilla; while a density of 0.64 g/cm^3^ can be expected in the anterior mandible, buccal shelf and midpalatal region.^25^ ^,^ ^26^ Therefore, the present findings reinforce the indication of SS-MIs instead of Ti-MIs for these insertion areas. However, it must be recognized that the artificial bone blocks used in the present study do not represent all the characteristics of specific regions. To do this, test blocks with different densities, representing both cancellous and cortical bone, should be prepared, in addition to working with different cortical thicknesses. In this way, the different regions of the bone, both interradicular and extra-alveolar, could be better represented. Thus, further research should investigate differences between the MIs evaluated in more specific representations of the different areas for the insertion of these MIs.

It is important to mention that some confounding factors may have influenced the present results. The risk of fracture during the clinical use of extra-alveolar MIs depends on other variables, such as the diameter and length of the MIs, geometric design, and insertion angle, in addition to the type of alloy chosen.^13^ ^-^ ^15^ The use of MIs from different brands implies possible variations in their geometric design. It has been previously demonstrated that even MIs with the same diameter but from different brands may present variations in their mechanical properties.^27^ Therefore, future studies should evaluate the interaction of all the mentioned factors on mechanical parameters of MIs inserted in bone of different densities.

## CONCLUSIONS

Considering the limitations of this *in vitro* study, the results seem to demonstrate that:

» Both SS-MIs and Ti-MIs provided adequate primary stability.
» Regardless of the type of MI material, MIs inserted in higher density bone had greater primary stability.
» SS-MIs showed greater flexural strength and less surface deformation than Ti-MIs, when inserted into high-density bone.

## References

[1] Kanomi R (1997). Mini-implant for orthodontic anchorage. J Clin Orthod.

[2] Chen Y, Kyung HM, Zhao WT, Yu WJ (2009). Critical factors for the success of orthodontic mini-implants a systematic review. Am J Orthod Dentofacial Orthop.

[3] Reynders R, Ronchi L, Bipat S (2009). Mini-implants in orthodontics: A systematic review of the literature. Am J Orthod Dentofacial Orthop.

[4] Sana S, Manjunath G (2013). Mini-implant materials an overview. IOSR J Dent Med Sci.

[5] Mecenas P, Espinosa DG, Cardoso PC, Normando D (2020). Stainless steel or titanium mini-implants A systematic review. Angle Orthod.

[6] Brown RN, Sexton BE, Chu TMG, Katona TR, Stewart KT, Kyung HM (2014). Comparison of stainless steel and titanium alloy orthodontic miniscrew implants a mechanical and histologic analysis. Am J Orthod Dentofacial Orthop.

[7] Gritsch K, Laroche N, Bonnet JM, Exbrayat P, Morgon L, Rabilloud M (2013). In vivo evaluation of immediately loaded stainless steel and titanium orthodontic screws in a growing bone. PLoS One.

[8] Garg H, Ahluwalia R, Grewal SB, Pandey SK, Mahesh A, Saini N (2022). Stainless steel vs titanium miniscrew implants: Evaluation of stability during retraction of maxillary and mandibular anterior teeth. J Orthodont Sci.

[9] Chang C, Liu SS, Roberts WE (2015). Primary failure rate for 1680 extra-alveolar mandibular buccal shelf mini-screws placed in movable mucosa or attached gingiva. Angle Orthod.

[10] Tsai CC, Chang HP, Pan CY, Chou ST, Tseng YC (2016). A prospective study of factors associated with orthodontic mini-implant survival. J Oral Sci.

[11] Pan CY, Chou ST, Tseng YC, Yang YH, Wu CY, Lan TH (2012). Influence of different implant materials on the primary stability of orthodontic mini -implants. Kaohsiung J Med Sci.

[12] Tseng Y, Ting C, Du J, Chen C, Wu J, Chen H (2016). Insertion torque, resonance frequency, and removal torque analysis of microimplants. Kaohsiung J Med Sci.

[13] Barros SE, Vanz V, Chiqueto K, Janson G, Ferreira E (2021). Mechanical strength of stainless steel and titanium alloy mini-implants with different diameters an experimental laboratory study. Prog Orthod.

[14] Scribante A, Montasser MA, Radwan ES, Bernardinelli L, Alcozer R, Gandini P (2018). Reliability of orthodontic miniscrews bending and maximum load of different Ti-6Al-4V titanium and stainless steel temporary anchorage devices (TADs). Materials (Basel).

[15] Vieira CA, Pires F, Hattori WT, de Araújo CA, Garcia-Junior MA, Zanetta-Barbosa D (2021). Structural resistance of orthodontic mini-screws inserted for extra-alveolar anchorage. Acta Odontol Latinoam.

[16] Krithikadatta J, Gopikrishina V, Datta M (2014). CRIS Guidelines (Checklist for Reporting In-vitro Studies) A concept note on the need for standardized guidelines for improving quality and transparency in reporting in-vitro studies in experimental dental research. J Conserv Dent.

[17] Devlin H, Horner K, Ledgerton D (1998). A comparison of maxillary and mandibular bone mineral densities. J Prosthet Dent.

[18] da Cunha AC, Marquezan M, Lima I, Lopes RT, Nojima LI, Sant'Anna EF (2015). Influence of bone architecture on the primary stability of different mini-implant designs. Am J Orthod Dentofacial Orthop.

[19] Ueda M, Matsuki M, Jacobsson M, Tjellström A (1991). Relationship between insertion torque and removal torque analyzed in fresh temporal bone. Int J Oral Maxillofac Implants.

[20] Kim S, Lee S, Cho I, Kim S, Kim T (2009). Rotational resistance of surface-treated mini-implants. Angle Orthod.

[21] Marquezan M, Mattos CT, Sant'Anna EF, Souza MMG, Maia LC (2014). Does cortical thickness influence the primary stability of miniscrews : A systematic review and meta-analysis. Angle Orthod.

[22] Motoyoshi M, Hirabayashi M, Uemura M, Shimizu N (2006). Recommended placement torque when tightening an orthodontic mini-implant. Clin Oral Implants Res.

[23] Meredith N (1998). Assessment of implant stability as a prognostic determinant. Int J Prosthodont.

[24] Nienkemper M, Handschel J, Drescher D (2014). Systematic review of mini-implant displacement under orthodontic loading. Int J Oral Sci.

[25] Misch CE (1989). Bone classification, training keys to implant success. Dent Today.

[26] Möhlhenrich SC, Heussen N, Modabber A, Bock A, Hölzle F, Wilmes B (2021). Influence of bone density, screw size and surgical procedure on orthodontic mini-implant placement - part B implant stability. Int J Oral Maxillofac Surg.

[27] Wilmes B, Panayotidis A, Drescher D (2011). Fracture resistance of orthodontic mini-implants a biomechanical in vitro study. Eur J Orthod.

